# Health Literacy and Oral Health Behaviors Among Dental Medicine Students: A Cross-Sectional Study

**DOI:** 10.3390/dj14070439

**Published:** 2026-07-15

**Authors:** Adina Oana Armencia, Roxana-Ionela Vasluianu, Andrei Nicolau, Monica Mihaela Scutariu, Alice Murariu, Irina Grădinaru, Laurian Lucian Francu, Anca Rapis, Irina Bamboi, Carina Ana-Maria Balcos

**Affiliations:** “Grigore T. Popa” University of Medicine and Pharmacy, 16 Universitatii Street, 700115 Iasi, Romania; adina.armencia@umfiasi.ro (A.O.A.); nicolau.andrei@umfiasi.ro (A.N.); alice.murariu@umfiasi.ro (A.M.); irina.gradinaru@umfiasi.ro (I.G.); laurianfrancu@yahoo.com (L.L.F.); anca.stupu@umfiasi.ro (A.R.); irina.bamboi@umfiasi.ro (I.B.); carina.balcos@umfiasi.ro (C.A.-M.B.)

**Keywords:** digital health, health behavior, health literacy, oral health, dental students

## Abstract

**Background/Objectives:** Health literacy is closely related to preventive behaviors, but its connection with actual oral health practices is still not fully clear, especially in the digital context. This study aimed to assess the association between general health literacy, digital oral health literacy, and oral health behaviors among dental medicine students. **Methods:** An observational cross-sectional study was conducted on a sample of 304 students. General health literacy was assessed using HLS-EU-Q16, digital oral health literacy using SMOHLQ, and oral health behaviors using HU-DBI. Statistical analyses included descriptive statistics, Spearman correlations, and linear regression models. **Results:** Oral health behaviors were moderate, while general literacy ranged from problematic to adequate, and digital literacy was high. No significant associations were identified between general health literacy and oral health behaviors (*p* > 0.05). Digital oral health literacy showed weak negative correlations with behaviors (r = −0.136, *p* = 0.018). Place of residence was a significant predictor (β = −0.394, *p* < 0.001), with the model explaining 16.2% of the variance. **Conclusions:** In this sample of dental medicine students, health literacy was not clearly related to better oral health behaviors. Digital oral health literacy was generally high, but its association with oral health behaviors was weak. Place of residence was associated with oral health behaviors, suggesting that preventive educational approaches for dental medicine students should also consider contextual factors.

## 1. Introduction

Health behaviors are major determinants of population health and result from the interaction of cognitive, social, and contextual factors. In recent decades, the increasing volume and diversity of health-related information have drawn attention to health literacy, defined as the ability to access, understand, appraise, and apply health information in decision-making [1,2]. Low health literacy has been associated with poorer health outcomes, reduced use of preventive services, and difficulties in adopting healthy behaviors, thereby contributing to persistent health inequalities [2,3]. However, health literacy does not refer only to how much health-related information a person has, but also involves understanding that information, judging whether it is relevant and reliable, and using it when making everyday decisions about health [1]. For this reason, the link between literacy and behavior may depend not only on knowledge itself, but also on how that knowledge is interpreted and put into practice.

In dentistry, these aspects are reflected in the concept of oral health literacy (OHL), which is considered important for the prevention and management of oral diseases. Dental caries and periodontal diseases remain among the most prevalent conditions worldwide, although they are largely preventable through appropriate behaviors [4]. Previous studies have shown that OHL may influence oral hygiene practices and the use of dental services [5,6,7,8]. At the same time, evidence suggests that this relationship is not always direct. Higher levels of literacy may improve understanding and attitudes toward prevention, but they do not consistently result in better oral health behaviors [9,10]. This indicates that oral health behaviors may also be shaped by social environment, access to resources, and behavioral norms.

Digitalization has further changed the way dental students seek and use health information. Digital health literacy extends traditional health literacy by including the ability to navigate, evaluate, and apply information obtained from online sources [11,12]. This aspect is particularly relevant among dental medicine students, who frequently use the Internet and social media for health-related information [13,14]. Although these platforms can provide rapid access to educational content, the quality of online information is highly variable and may include incomplete, inaccurate, or commercially driven content [15,16,17].

In the field of oral health, social media may act both as a tool for health promotion and as a source of misinformation, influencing users’ perceptions, decisions, and behaviors [18,19,20]. Similarly, digital interventions, including eHealth and mHealth applications, have been shown to improve knowledge, but their effects on behavioral change remain inconsistent and context-dependent [21,22]. This suggests that access to information alone is not sufficient to produce behavioral change. Motivation, self-efficacy, social support, and other psychosocial factors may also contribute to the translation of knowledge into practice [23,24].

Oral health behaviors are directly involved in the onset and progression of oral diseases and may influence the long-term need for restorative and rehabilitative treatments. Therefore, identifying the factors associated with these behaviors, including general and digital dimensions of health literacy, is relevant for prevention and reducing the burden of dental care.

Dental medicine students are an important group in which to study oral health behaviors, because they receive training related to oral diseases, prevention, and patient education. Therefore, their level of knowledge and literacy may be expected to influence their own oral health practices. Steinvik et al. reported that among dental students, higher health literacy was associated with more frequent use of dental services and better self-rated oral health [25]. Similarly, Wang et al. showed that students from medical fields had more favorable oral health behaviors than non-medical students, including toothbrushing habits and the use of oral hygiene aids [26]. However, knowledge acquired during training is not always reflected in daily behavior. For this reason, dental medicine students should be examined separately, especially because they are future oral health professionals and will later have a role in prevention and patient counseling.

This study aimed to examine the relationship between general health literacy, digital oral health literacy, and oral health behaviors among dental medicine students. The study also explored whether these relationships are influenced by contextual factors (particularly place of residence) within this population.

Based on previous evidence, it was hypothesized that among dental medicine students, higher levels of health literacy would be associated with more favorable oral health behaviors. It was also hypothesized that digital oral health literacy would show a closer relationship with oral health behaviors than general health literacy, and that these associations would vary according to contextual factors, particularly place of residence.

## 2. Materials and Methods

### 2.1. Study Design

The study was designed as an observational cross-sectional study to examine the relationships among digital health literacy, oral health literacy, and oral health behaviors in dental medicine students. Data were collected at a single time point, enabling the assessment of associations between the variables of interest and the exploration of contextual factors, such as place of residence.

### 2.2. Participants and Selection Criteria

The study was conducted in March 2026 among students from the Faculty of Dental Medicine of the “Grigore T. Popa” University of Medicine and Pharmacy in Iași, following approval from the institution’s Ethics Committee (No. 718/22.02.2026). The sample size was estimated for a cross-sectional design assuming a 95% confidence level and a 5% margin of error [27], resulting in a minimum required sample of 205 participants.

Eligible participants were students enrolled at the Faculty of Dental Medicine during the study period, aged between 20 and 30 years, who were able to complete the questionnaire and agreed to take part in the study. The exclusion criteria included incomplete questionnaires, duplicate responses, missing data, and absence of valid informed consent. Students were invited through an online announcement distributed via student communication groups and academic channels. Before completing the questionnaire, they were informed during the practical training sessions about the purpose of the study, the voluntary nature of participation, confidentiality, anonymity, and the use of the collected data. A total of 421 eligible students were invited to participate in the study. Based on the sample size calculation, the minimum required number of participants was 205. Students completed the questionnaire only after agreeing to participate and providing informed consent.

**Figure 1:**
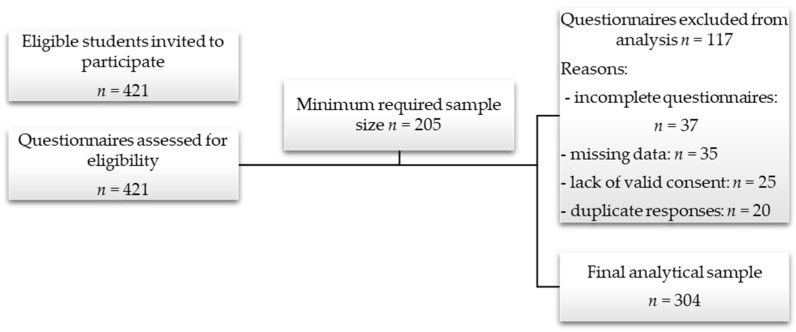
Participant recruitment flow diagram. *n* indicates the number of participants.

Overall, 421 questionnaires were assessed for eligibility. Of these, 117 were excluded from the final analysis: 37 because of incomplete responses, 35 because of missing data, 25 because valid informed consent was not available, and 20 due to duplicate responses. After applying the inclusion and exclusion criteria, the final analytical sample consisted of 304 respondents, which exceeded the minimum required sample size (Figure 1).

Since the study followed a voluntary response-based cross-sectional design, no randomization procedure was applied.

### 2.3. Research Instrument

#### 2.3.1. HLS-EU-Q16

General health literacy was assessed using the short version of the European Health Literacy Survey (HLS-EU-Q16), developed within the European Health Literacy Survey (HLS-EU) project as a condensed form of the original instrument [28]. The questionnaire measures individuals’ ability to access, understand, appraise, and apply health-related information across healthcare, disease prevention, and health promotion contexts.

The instrument consists of 16 items that cover key competencies in health information processing. Responses are recorded on a four-point Likert scale ranging from “very difficult” to “very easy”.

For analysis, responses were dichotomized, with categories indicating difficulty coded as 0 and those indicating ease coded as 1. The total score was calculated by summing item responses, resulting in scores ranging from 0 to 16, with higher values indicating higher levels of health literacy.

For interpretation, the HLS-EU-Q16 scores were grouped using the scoring categories recommended for this instrument. After dichotomizing the answers, the total score ranged from 0 to 16 and indicates inadequate health literacy for scores of 0–8, problematic health literacy for scores of 9–12, and adequate health literacy for scores of 13–16 [29].

Validation studies conducted in the Romanian population have demonstrated good internal consistency of the instrument, with a Cronbach’s alpha of approximately 0.84, supporting its use in assessing health literacy [29].

#### 2.3.2. Social Media Oral Health Literacy Questionnaire (SMOHLQ)

The Social Media Oral Health Literacy Questionnaire (SMOHLQ) was used to assess oral health literacy in the digital environment. It focuses on how individuals access, understand, critically evaluate, and use oral health information available online. The instrument is relatively recent in the literature and was developed to capture specific aspects of digital literacy in the context of social media use [30,31].

The questionnaire consists of 27 items, organized into three main dimensions, which reflect the stages of information processing in the digital environment. The first dimension, access and comprehension (items 1–10), targets the participants’ ability to identify, find, and interpret oral health information available online. The second dimension, critical appraisal (items 11–21), assesses the ability to evaluate the credibility of sources, to differentiate valid information from commercial or misleading content, and to compare information from different sources. The third dimension, behavioral impact (items 22–27), measures the extent to which the accessed information influences behaviors and decisions related to oral health.

The items are formulated as self-reported statements and are rated on a 5-point Likert scale, from 1 (“strongly disagree”) to 5 (“strongly agree”). Higher values indicate a higher level of oral health literacy in the digital environment. A mean score is calculated for each dimension, and the total score is obtained by aggregating responses across the entire questionnaire. The multidimensional structure of the instrument allows for the separate analysis of cognitive, evaluative, and behavioral components. In the analysis process, items that did not directly contribute to the calculation of the subscale were excluded from the dimensional assessment. The version used in the study was translated and culturally adapted into Romanian, following the standard translation and backtranslation steps, with verification of semantic and conceptual equivalence [32]. The final form was pre-tested for clarity and comprehensibility before application. The SMOHLQ questionnaire presented good internal consistency, with a Cronbach’s alpha coefficient of 0.856 for the total scale and 0.873 for the standardized version.

#### 2.3.3. Hiroshima University Dental Behavior Inventory

Oral health behaviors were assessed using the Hiroshima University Dental Behavior Inventory (HU-DBI), an instrument developed by Kawamura for assessing oral hygiene habits and attitudes toward maintaining dental health [33].

The HU-DBI consists of 20 statements related to oral hygiene behaviors, attitudes, and self-perceived oral health, including toothbrushing habits, use of hygiene aids, gingival bleeding, dental plaque, and preventive attitudes. Participants answered each item by indicating agreement or disagreement.

The total HU-DBI score was calculated based on the 12 standard scored items: 2, 4, 6, 8, 9, 10, 11, 12, 14, 15, 16, and 19. According to the original scoring system, one point was assigned for each response considered favorable to oral health. Affirmative responses were scored for items 4, 9, 11, 12, 16, and 19, whereas negative responses were scored for items 2, 6, 8, 10, 14, and 15. After recoding, all scored items had the same direction, with 1 representing a favorable response and 0 an unfavorable response. The final score ranged from 0 to 12, with higher scores indicating more favorable oral health behaviors.

In the present sample, reliability of the 12 scored HU-DBI items was assessed after recoding. Internal consistency was evaluated using Cronbach’s alpha, and reliability was also examined using the intraclass correlation coefficient.

### 2.4. Statistical Analysis

Statistical analysis was conducted using SPSS software (version 26.0; IBM Corp., Armonk, NY, USA).

Descriptive statistics were used to summarize the characteristics of the sample and the main variables, including mean values, standard deviations, and observed ranges.

Since the variables did not follow a normal distribution, non-parametric tests were applied. Group comparisons were carried out using the Mann–Whitney U test or the Kruskal–Wallis test, depending on the number of groups.

Associations between variables were examined using Spearman correlation coefficients, with their strength interpreted according to standard conventions.

Linear regression analysis was performed to identify factors associated with oral health behaviors and to estimate the contribution of the independent variables to the HU-DBI score. To explore potential interaction effects, the SMOHL_access variable was centered around its mean, and an interaction term with the environment of origin was included in the model.

Gender and environment of origin were treated as binary variables in the analysis. Multicollinearity was checked using variance inflation factors (VIFs). The variables included in the regression models were selected in relation to the aim of the study. General health literacy, the dimensions of digital oral health literacy, gender, and place of residence were entered into the models because they were relevant to the proposed hypotheses and to possible differences in oral health behaviors. Gender and place of residence were coded as binary variables. Place of residence was self-reported by participants and coded as urban or rural. Students living in a city or municipality were included in the urban group, while those living in a village or commune were included in the rural group.

The regression models were checked before interpretation. Linearity, residual distribution, homoscedasticity, and possible influential cases were assessed using residual plots and standard diagnostic measures. Multicollinearity was evaluated using variance inflation factors, and all VIF values were below 2. Missing data were not imputed, as only fully completed questionnaires were included in the final analysis. Gender and place of residence were retained in the models as potential confounding variables. The variable was then used as a binary variable in the statistical analysis.

A *p*-value of less than 0.05 was considered statistically significant in all analyses.

## 3. Results

### 3.1. General Characteristics

The study group included 304 participants, most of whom were female (61.8%), while males accounted for 38.2%. More than half of the respondents were from rural areas (56.9%) compared to 43.1% from urban areas. Regarding age distribution, most participants were between 20 and 24 years old (53.3%), followed by those aged 25 to 30 years (38.8%), while only a small proportion (7.9%) were over 30 years old (Table 1).

### 3.2. Validation of the HU-DBI Questionnaire

The internal reliability of the questionnaire was good, with a Cronbach’s alpha coefficient of 0.816 for the 12 items analyzed. The same value was obtained for the alpha calculation on the standardized items, suggesting that the items are relatively homogeneous and contribute in a balanced manner to the assessment of the intended construct. These results support the use of the instrument in subsequent analyses (Table 2).

The mean values of the items ranged from 1.34 to 1.78, and the standard deviations ranged from 0.41 to 0.50. The lowest means were observed for the items regarding checking the teeth in the mirror after brushing (M = 1.34; SD = 0.47) and carefully brushing each tooth (M = 1.35; SD = 0.48). The highest mean values were recorded for the items regarding the possibility of cleaning the teeth without toothpaste (M = 1.78; SD = 0.41), sometimes too much time given to brushing (M = 1.76; SD = 0.43) and the lack of professional training on brushing technique (M = 1.75; SD = 0.43).

The relatively close standard deviations between items indicate a low variability of responses within the sample, which is consistent with the good level of internal consistency obtained for the questionnaire (Cronbach α = 0.816). Item-level descriptive statistics are presented in Supplementary Table S1.

The corrected item-total correlations ranged from 0.357 to 0.545, exceeding in all cases the minimum recommended threshold of 0.30. The highest correlation was recorded for the item “I think my teeth are getting worse despite my daily brushing” (r = 0.545), and the lowest for the item “I have never been professionally taught how to brush” (r = 0.357). The Cronbach’s alpha of item deleted values ranged from 0.796 to 0.812 without exceeding the overall value of the scale (α = 0.816). Therefore, the removal of any item would not have improved the reliability of the instrument, which supports the preservation of the original structure of the questionnaire. Detailed data are presented in Supplementary Table S2.

The intraclass correlation coefficient was calculated using the two-way mixed effects model, using the definition of consistency. For the mean measures, the ICC value was 0.816 (95% CI: 0.784–0.845; *p* < 0.001), indicating good reliability of the total score of the instrument. The ICC for the individual measures was lower at 0.270 (95% CI: 0.233–0.313), which can be explained by the natural variability between items. The F test confirmed that the level of agreement observed was statistically significant (F = 5.446; *p* < 0.001) (Table 3).

To check whether the items could be factor analyzed, the Kaiser–Meyer–Olkin (KMO) coefficient and the Bartlett test of sphericity were used. The KMO value was 0.721, indicating that the sample was suitable for exploratory factor analysis. The Bartlett test was statistically significant (χ^2^ = 1309.112; df = 66; *p* < 0.001), which shows that there were sufficient correlations between the items to explore the factor structure of the questionnaire (Table 4).

Exploratory factor analysis, performed by principal component analysis, identified four components with eigenvalues greater than 1, according to the Kaiser criterion. The first component had the highest contribution, with an eigenvalue of 4.005 and 33.38% of the total variance explained. The following components explained 14.69%, 11.41%, and 8.95%, respectively, of the variance. Together, the four components explained 68.43% of the total variance of the instrument. After Varimax rotation, the distribution of variance between the factors became more balanced, with each component explaining between 14.46% and 19.76% of the variance. The complete data are presented in Supplementary Table S3.

The scree plot, presented in Supplementary Figure S1, supported the retention of four factors, with a visible inflection point after the fourth component. This result was consistent with the Kaiser criterion, which indicated four components with eigenvalues greater than 1.

Exploratory factor analysis with Varimax rotation indicated a four-component structure, which together explained 68.43% of the total variance. The first factor brought together items related to oral hygiene monitoring and evaluation, and the second included items related to oral health perception and health education. The third factor was associated with attitudes regarding prevention and use of dental services, while the fourth reflected perceptions related to brushing techniques and oral hygiene efficiency. This distribution of items supports the construct validity of the HU-DBI questionnaire in the analyzed sample. The complete matrix of factor loadings is presented in Supplementary Table S4.

### 3.3. Descriptive Statistics of Health Literacy and Oral Health Behaviors

The descriptive results show that oral health behaviors (HU-DBI) were moderate on average (7.61 ± 1.94 out of a maximum of 12), indicating that some appropriate practices were present, but not consistently across participants (Table 5).

Regarding general health literacy, the mean HLS-EU-Q16_total score was 10.74 ± 4.04 (out of 16), corresponding to a level between problematic and adequate. The relatively high variability suggests differences between respondents in their ability to understand and use health information.

For oral health literacy in a digital context, the SMOHL_total scores were generally high (4.04 ± 0.33 out of 5). Dimensional analysis showed the highest scores for the critical component (4.54 ± 0.40), followed by access to information (4.20 ± 0.36). In contrast, the behavioral dimension had lower values (2.87 ± 0.69), suggesting difficulties in applying information in practice.

Overall, the results suggest a difference between knowledge-related skills and actual behaviors, with participants showing stronger abilities in accessing and evaluating information than in applying it in everyday life (Table 5).

### 3.4. Correlation Analysis

Spearman correlation analysis did not show significant associations between general health literacy (HLS-EU-Q16) and the other variables analyzed. The total health literacy score was not correlated with either digital oral health literacy or oral health behaviors (*p* > 0.05) (Table 6).

Within the SMOHL instrument, the dimensions were correlated with each other. The total score showed strong positive correlations with access to information (r = 0.764, *p* < 0.001) and the critical component (r = 0.780, *p* < 0.001), and a moderate correlation with the behavioral dimension (r = 0.606, *p* < 0.001). Access to information was also correlated with the critical component (r = 0.708, *p* < 0.001).

Weak negative correlations were observed in relation to oral health behaviors. The total SMOHL score (r = −0.136, *p* = 0.018) and the behavioral dimension (r = −0.128, *p* = 0.026) were both associated with the HU-DBI score, although the strength of these associations was low.

The relationships between variables were also analyzed separately according to the environment of origin. In urban areas, no significant correlations were observed between oral health literacy and oral health behaviors. The only statistically significant association was between the behavioral dimension of SMOHL and HU-DBI (r = −0.234, *p* = 0.007), although the correlation was weak (Table 7).

In rural areas, some weak negative correlations were identified. The total SMOHL score was negatively correlated with HU-DBI (r = −0.232, *p* = 0.002), and a similar pattern was observed for the access component (r = −0.210, *p* = 0.005).

In both groups, general health literacy was not associated with oral health behaviors (*p* > 0.05).

The regression model reached statistical significance (F = 9.593, *p* < 0.001) and accounted for 16.2% of the variance in oral health behaviors (R^2^ = 0.162; adjusted R^2^ = 0.145).

Among the included predictors, only the access dimension of digital oral health literacy showed a significant negative association with oral health behaviors (B = −0.851, β = −0.159, 95% CI: −1.644 to −0.058, *p* = 0.035). Place of residence also emerged as a strong and significant predictor (B = −1.539, β = −0.394, 95% CI: −1.991 to −1.086, *p* < 0.001), suggesting that place of residence may be related to oral health behaviors in this student sample.

In contrast, general health literacy, the critical appraisal component, the behavioral dimension of digital literacy, and gender were not significantly associated with oral health behaviors (*p* > 0.05).

No issues related to multicollinearity were identified, with all VIF values remaining below 2 (Table 8).

An interaction model was further tested to explore whether the association between access to information and oral health behaviors varied according to place of residence. The model explained 17.6% of the variance in oral health behaviors (R^2^ = 0.176; adjusted R^2^ = 0.165).

A significant interaction effect was observed between access to information and place of residence (B = −1.879, β = −0.596, 95% CI: −3.211 to −0.547, *p* = 0.006), suggesting that the relationship between digital oral health literacy and oral health behaviors differs between urban and rural participants. Place of residence remained a significant predictor (B = −1.511, β = −0.387, 95% CI: −1.955 to −1.066, *p* < 0.001). In contrast, the main effect of access to information was not statistically significant (B = 1.900, β = 0.354, 95% CI: −0.396 to 4.196, *p* = 0.104), and no significant association was found for gender (*p* = 0.625) (Table 9).

To better understand this pattern, the analysis was also carried out separately for urban and rural participants. In the rural group, access to information was significantly associated with oral health behaviors, with higher levels of access linked to less favorable behaviors (B = −1.663, β = −0.316, 95% CI: −2.451 to −0.875, *p* < 0.001). In contrast, no significant association was observed in the urban group (B = 0.275, β = 0.047, 95% CI: −0.768 to 1.318, *p* = 0.603).

Gender was significantly associated with oral health behaviors in the urban group (B = 0.639, β = 0.188, 95% CI: 0.029 to 1.249, *p* = 0.040), but not in the rural group (B = −0.356, β = −0.087, 95% CI: −0.969 to 0.256, *p* = 0.253) (Table 10).

Taken together, these findings are consistent with the interaction model and suggest that the relationship between digital oral health literacy and oral health behaviors is shaped by the environment of origin.

## 4. Discussion

In the present sample of dental medicine students, health literacy was not clearly reflected in oral health behaviors. Although the students had relatively high levels of general and digital literacy, these levels were not consistently associated with better oral health behavior scores. This may indicate that even among students exposed to oral health information during their training, knowledge and literacy are not always translated into daily practice.

Evidence from the literature indicates that the link between health literacy and health behaviors is not necessarily direct [1,34]. Current conceptual frameworks describe health literacy as a multidimensional construct, encompassing a range of competencies from understanding information to applying it in practice [35]. In addition, the adoption of health behaviors is influenced by organizational and structural factors, not only by individual abilities to access and process information [36,37,38].

Recent studies have also highlighted the role of health systems in supporting health literacy, particularly through organizational strategies and by adapting communication to patients’ needs [39]. At the same time, exposure to information in digital environments does not ensure its appropriate use, especially given the variability in the quality of online sources [38,39,40,41,42,43,44].

A first relevant finding was the lack of a significant association between general health literacy and oral health behaviors in this sample of dental medicine students. This result differs from some previous studies that reported a positive relationship between health literacy and preventive behaviors. For example, Steinvik et al. found that among dental medicine students in Norway, higher health literacy was associated with more frequent use of dental services and better self-rated oral health [25]. However, other studies have shown that the relationship between oral health literacy and oral health behaviors may differ according to the educational context and the characteristics of the studied group [26,45]. In our study, the use of a general health literacy instrument, such as the HLS-EU-Q16, may partly explain the absence of a clear association with HU-DBI scores.

In addition, the relatively low proportion of variance explained by the regression model indicates that oral health behaviors are influenced by multiple factors, with health literacy representing only one component. This observation is in line with previous studies emphasizing the contribution of contextual, social, and structural determinants in shaping health-related behaviors.

These results can be compared with studies carried out in students or similar educational groups. Wang et al. reported that students from medical fields had more favorable oral health behaviors than non-medical students, including more frequent toothbrushing and the use of oral hygiene aids [26]. In our study, however, digital oral health literacy was only weakly associated with oral health behaviors. Thus, among dental medicine students, access to oral health information and the ability to evaluate it were not clearly reflected in consistent preventive practices. Other studies have also reported that the relationship between oral health literacy and oral health behaviors may vary according to educational context and the characteristics of the studied group [45,46,47].

The mean HU-DBI score in the present study was 7.61 ± 1.94, indicating a moderate level of oral health behavior among dental medicine students. This value is comparable to the pooled mean reported in a recent systematic review and meta-analysis of dental students, where the overall HU-DBI score was 7.15, with a 95% confidence interval of 6.82–7.47 [48]. It is also close to values reported in other student populations. For example, a study conducted among dental students in Palestine found a mean HU-DBI score of 7.23 ± 1.77, with higher scores among female students and those in clinical years [49]. Similar patterns have also been described among Romanian dental students, where oral self-care behaviors differed according to sex and year of study [50]. Taken together, these findings suggest that dental education may contribute to better preventive awareness, but it does not necessarily lead to consistently optimal oral self-care practices.

A relevant aspect that emerges from these findings is that the link between digital oral health literacy and oral health behaviors does not appear to be consistent across contexts. Although the overall analysis pointed to weak or inconsistent associations, the interaction model and the stratified analyses indicate a more differentiated pattern.

More specifically, access to information was associated with oral health behaviors only in the rural group, where higher levels of access were linked to less favorable behaviors. In contrast, no meaningful association was identified among urban participants. This suggests that the influence of digital literacy may be shaped by the context in which information is accessed and used, rather than acting independently.

In our study, the association between access to digital oral health information and oral health behaviors was found mainly among students from rural areas. This may mean that the students’ background influences how oral health information is used in daily life. For students from rural areas, greater access to online information may not be enough if previous habits, access to dental care, or family and social routines differ from those of students from urban areas. Among students from urban areas, access to information was not significantly associated with oral health behaviors, which suggests that other factors may be more important in this subgroup.

Taken together, these results show that digital oral health literacy should be considered in relation to the students’ background. In this sample, its association with oral health behaviors was weak and varied according to place of residence. Thus, for dental medicine students, access to oral health information alone may not be enough if educational approaches do not also focus on how this information is used in daily routines.

A particularly relevant aspect of the present findings is the difference observed be-tween the components of digital oral health literacy. Participants scored higher on the access and critical appraisal dimensions than on the behavioral component. This pattern suggests that the ability to locate and interpret information does not automatically translate into its application in everyday routines. Evidence from the literature supports this distinction. A recent systematic review of mHealth and eHealth interventions showed that although such tools can improve knowledge, their impact on behavior is less consistent and depends on factors such as intervention design and user engagement [21,23]. Similar patterns have been reported in other studies, where the cognitive dimension tends to be more developed than the behavioral one, even among medical students [48].

This gap between knowledge and behavior has also been noted in previous HU-DBI studies. In a multinational study of Arab dental students, oral health behaviors differed between countries and were influenced by both sociodemographic and educational factors [51]. Similar findings were reported in a 17-country study of final-year dental students, which showed important cross-cultural differences in oral health attitudes and practices [52]. In this context, the results of the present study should not be seen as an isolated finding. Rather, they reflect a broader pattern in which dental knowledge and professional training do not act alone, but are shaped by cultural background, local educational norms, and the social context in which students develop their preventive habits.

The negative correlations observed between oral health literacy and oral health behaviors, although weak, require careful consideration. One possible explanation relates to the use of self-reported measures, where students with higher literacy levels may adopt a more critical perspective on their own behaviors. In this context, higher literacy may be associated with increased awareness of the gap between recommended practices and actual habits, without necessarily indicating poorer behaviors [21].

In this group of dental medicine students, the inverse associations between digital oral health literacy and oral health behaviors should be viewed with caution. Although significant, these associations were weak and have limited practical relevance. The cross-sectional design also does not allow for the direction of the relationship to be established. Other factors that were not fully assessed, such as access to dental care, previous dental experiences, motivation, social norms, or family routines, may also have influenced the results. Another possible explanation is that students with higher literacy may judge their own behaviors more critically, which could lead to less favorable self-reports.

The environment of origin also appears to play an important role. Stratified analyses and the interaction model showed that the relationship between access to information and oral health behaviors differs between urban and rural areas. This finding is in line with previous research highlighting the influence of social and structural factors on health behaviors [1]. Digital health interventions have been proposed as a way to reduce inequalities, but their effectiveness depends on access to resources and on the ability to translate information into action [23]. For students from rural areas, knowledge may be harder to apply in daily life because of access to dental care, previous habits, or family and social routines, even when literacy levels are adequate [53,54].

The pattern observed among rural students in the present study is in line with previous evidence suggesting that digital health behaviors depend not only on access to information, but also on the wider context in which this information is used. Ji et al. showed that among rural residents, digital literacy was an important predictor of engagement in digital health behaviors, while the role of health literacy varied depending on the type of behavior analyzed [53]. In the field of oral health, Iyer et al. also reported a significant association between eHealth literacy and oral health behavior among dental outpatients. However, their study further showed that eHealth literacy was influenced by factors such as age, income, and education [55]. These findings support the idea that digital access and perceived literacy should not be interpreted in isolation. They need to be considered together with socioeconomic background and environmental constraints, particularly when differences between rural and urban groups are observed.

The significance of background as a predictor in the regression model further supports this interpretation. Rather than acting independently, health literacy appears to operate in interaction with contextual factors. This view is consistent with Nutbeam’s model, which conceptualizes health literacy as a multidimensional construct shaped by both individual competencies and environmental demands [34].

At the same time, the absence of significant gender differences and the lack of variation in HU-DBI scores across health literacy categories point to the multifactorial nature of oral health behaviors. Evidence from studies conducted in university populations indicates that educational context, clinical exposure, and social norms may play a more prominent role than general health literacy alone [41,48].

From an educational perspective, oral health information alone may not be enough to improve self-reported oral health behaviors among dental medicine students. Even though these students are exposed to dental knowledge during training, the way they apply this knowledge in daily life may depend on motivation, previous habits, and their social or educational background. For this reason, preventive education in dental curricula should not be limited to providing information, but should also address behavior, habits, and the use of oral hygiene recommendations in students’ own routines.

### 4.1. Strengths of the Study

One strength of this study lies in the analysis of multiple dimensions of literacy, including both general health literacy and oral health literacy in a digital context. This approach made it possible to explore in more detail the relationship between access to in-formation and health behaviors.

The use of validated instruments, such as the Romanian version of the HLS-EU-Q16, represents another advantage, ensuring a consistent and comparable assessment of health literacy.

An additional strength is the inclusion of the environment of origin in the analysis, along with testing interactions between variables. This allowed for the identification of differences that might not have been apparent in an overall analysis.

### 4.2. Study Limitations

Several limitations should be considered when interpreting the results. First, the cross-sectional design does not allow causal relationships to be established between health literacy and oral health behaviors, but only associations.

Second, the data were collected using self-reported questionnaires, which may have influenced the accuracy of the responses. This aspect is important in the present sample, as dental medicine students are familiar with recommended oral health behaviors and may tend to report practices that are considered appropriate. Therefore, the HU-DBI scores may not fully reflect everyday oral hygiene behaviors.

In this study, no additional psychometric evaluation of the HU-DBI was performed on the investigated sample. Nevertheless, the instrument is widely used and has demonstrated adequate psychometric properties across various cultural contexts, allowing comparison with previous research. Further assessments of internal consistency and validity could, however, strengthen the interpretation of the findings in this population.

Another limitation is the absence of objective clinical indicators of oral health, such as dental or periodontal status, which limits a direct comparison with clinical outcomes. The findings should be interpreted in relation to the study sample. Participants were recruited from a single institution and were dental medicine students, which may have influenced both their level of health literacy and their reported oral health behaviors. For this reason, the results may not be directly generalizable to all dental medicine students, particularly to students from other educational settings or to non-student populations.

It should also be noted that the regression model explained only a relatively small proportion of the variance in oral health behaviors, suggesting that additional factors not included in the analysis, such as socioeconomic conditions or access to services, may play a role.

Finally, the assessment of digital oral health literacy was based on the participants’ self-perception of their skills, which may not fully reflect how information is used in practice.

### 4.3. Future Directions

Further research could examine the relationship between health literacy and behaviors over time, using longitudinal designs that would allow for a better understanding of how these dimensions evolve. The inclusion of clinical indicators could also provide a clearer link between literacy levels and actual oral health status.

It may also be relevant to explore additional factors that could influence this relationship, such as self-efficacy, motivation, or trust in information sources, particularly in the context of the frequent use of digital media. The findings also point to the importance of the environment of origin, suggesting that future interventions should take local context into account. This may be especially relevant in rural areas, where access to resources and the practical use of information can be more limited.

Another direction would be to focus not only on access to information, but also on how it is applied in practice. Studies evaluating digital interventions aimed at supporting behavioral change, rather than only increasing knowledge, could provide valuable insights.

Overall, these findings highlight the need to reconsider how health literacy is approached, with greater emphasis on its role in facilitating behavioral change in real-life settings.

## 5. Conclusions

In the present study, general health literacy was not associated with oral health behaviors among dental medicine students. Digital oral health literacy showed only weak relationships with these behaviors, suggesting that better access to oral health information online does not necessarily mean better daily oral hygiene practices.

Place of residence appeared to be an important factor. The association between access to digital oral health information and oral health behaviors was different in urban and rural participants, which suggests that the context in which dental medicine students live may influence how health information is used in practice.

Overall, the results indicate that in this sample, oral health behaviors cannot be explained by literacy or access to information alone. Preventive approaches aimed at dental medicine students should also take into account contextual factors that may affect the application of oral health information in everyday life.

## Figures and Tables

**Table 1:** Sociodemographic characteristics of the study population.

Variable	*n* (%)
Gender	
Male	116 (38.2%)
Female	188 (61.8%)
Place of residence	
Urban	131 (43.1%)
Rural	173 (56.9%)
Age group	
20–24 years	162 (53.3%)
25–30 years	118 (38.8%)
Over 30 years	24 (7.9%)

**Table 2:** Reliability indices of the HU-DBI questionnaire.

Cronbach’s Alpha	Standardized Cronbach’s Alpha	No. of Items
0.816	0.816	12

**Table 3:** Intraclass correlation coefficient analysis of the HU-DBI questionnaire.

Measure	ICC	95% CI	F	df1	df2	*p*-Value
Single measures	0.270	0.233–0.313	5.446	303	3333	<0.001
Average measures	0.816	0.784–0.845	5.446	303	3333	<0.001

Two-way mixed-effects model with consistency definition.

**Table 4:** KMO and Bartlett’s test for exploratory factor analysis.

KMO	Bartlett’s Test χ^2^	df	*p*-Value
0.721	1309.112	66	<0.001

KMO = Kaiser–Meyer–Olkin measure of sampling adequacy.

**Table 5:** Descriptive statistics of the studied variables.

Variable	N	Min	Max	Mean ± SD
HU-DBI_total	304	4.00	11.00	7.61 ± 1.94
HLS-EU-Q16_total	304	2.00	16.00	10.74 ± 4.04
SMOHL_total	304	3.19	4.67	4.04 ± 0.33
SMOHL_access	304	3.30	4.80	4.20 ± 0.36
SMOHL_critical	304	3.55	5.00	4.54 ± 0.40
SMOHL_behavior	304	1.67	4.33	2.87 ± 0.69

**Table 6:** Spearman correlations between the studied variables.

Variable	1	2	3	4	5	6
1. SMOHL_total	1					
2. SMOHL_access	0.764 **	1				
3. SMOHL_critical	0.780 **	0.708 **	1			
4. SMOHL_behavior	0.606 **	0.173 **	0.057	1		
5. HL_total	0.052	0.035	0.030	0.021	1	
6. HU-DBI_total	−0.136 *	0.021	−0.017	−0.128 *	0.007	1

* *p* < 0.05, ** *p* < 0.01.

**Table 7:** Spearman correlations between variables, according to the environment of origin.

Variable	Urban (r)	Rural (r)
SMOHL_total—HU-DBI	−0.131	−0.232 **
SMOHL_access—HU-DBI	0.051	−0.210 **
SMOHL_critical—HU-DBI	−0.046	−0.019
SMOHL_behavior—HU-DBI	−0.234 **	−0.053
HLS-EU-Q16_total—HU-DBI	0.087	−0.015

** *p* < 0.01.

**Table 8:** Predictors of oral health behaviors.

Variable	B	SE	β	95% CI for B	*p*
HLS-EU-Q16_total	0.007	0.026	0.015	−0.043 to 0.057	0.784
SMOHLQ_Access	−0.851	0.403	−0.159	−1.644 to −0.058	0.035 *
SMOHLQ_Critical	−0.353	0.338	−0.072	−1.019 to 0.313	0.298
SMOHLQ_Behavior	−0.200	0.157	−0.072	−0.509 to 0.108	0.202
Gender	−0.109	0.223	−0.027	−0.548 to 0.330	0.626
Residence	−1.539	0.230	−0.394	−1.991 to −1.086	<0.001 **

B = unstandardized coefficient; SE = standard error; β = standardized coefficient. * *p* < 0.05, ** *p* < 0.01.

**Table 9:** Interaction model predicting oral health behaviors (HU-DBI score).

Variable	B	SE	β	95% CI for B	*p*
Residence	−1.511	0.226	−0.387	−1.955 to −1.066	<0.001 **
Gender	0.108	0.221	0.027	−0.327 to 0.544	0.625
SMOHL_Access centered	1.900	1.167	0.354	−0.396 to 4.196	0.104
SMOHL_Access × Residence	−1.879	0.677	−0.596	−3.211 to −0.547	0.006 **

B = unstandardized coefficient; SE = standard error; β = standardized coefficient. ** *p* < 0.01.

**Table 10:** Linear regression models stratified by environment of origin.

Group	Variable	B	SE	β	95% CI for B	*p*
Urban	Gender	0.639	0.308	0.188	0.029 to 1.249	0.040 *
Urban	SMOHLQ_Access	0.275	0.527	0.047	−0.768 to 1.318	0.603
Rural	Gender	−0.356	0.310	−0.087	−0.969 to 0.256	0.253
Rural	SMOHLQ_Access	−1.663	0.399	−0.316	−2.451 to −0.875	<0.001 **

β = standardized coefficient; * *p* < 0.05, ** *p* < 0.01.

## Data Availability

The data supporting the findings of this study are available from the corresponding author upon reasonable request.

## Supplementary Materials

The following supporting information can be downloaded at: https://www.mdpi.com/article/10.3390/dj14070439/s1, Table S1: Item-level descriptive statistics of the HU-DBI questionnaire; Table S2: Item-total statistics of the HU-DBI questionnaire; Table S3: Total variance explained in the exploratory factor analysis of the HU-DBI questionnaire; Figure S1. Scree plot for the exploratory factor analysis of the HU-DBI questionnaire; Table S4: Rotated component matrix of the HU-DBI questionnaire.

## Abbreviations

The following abbreviations are used in this manuscript:

HLS-EU-Q16	European Health Literacy Survey
SMOHLQ	Social Media Oral Health Literacy Questionnaire
HU-DBI	Hiroshima University Dental Behavior Inventory
*p*	*p*-Value
r	Correlation coefficient
β	Standardized regression coefficient
OHL	Oral health literacy
HLS-EU	European Health Literacy Survey
SPSS	Statistical Package for the Social Sciences
Min	Minimum
Max	Maximum
SD	Standard Deviation
F	F-statistic
SE	Standard Error
B	Regression coefficient (unstandardized coefficient)
