# Supplementary material for: Health Literacy and Oral Health Behaviors Among Dental Medicine Students: A Cross-Sectional Study
Source: Dent J (Basel). 2026 Jul 15;14(7):439. doi: 10.3390/dj14070439 (PMC13408259; doi:10.3390/dj14070439)
Supplement: Supplementary file 1 [file dentistry-14-00439-s001.zip › dentistry-4334537-supplementary.pdf]

Supplementary Table S1. Item-level descriptive statistics of the HU-DBI questionnaire

Item Statistics			
	Mean	Std. Deviation	N
My gums tend to bleed when I brush my teeth	1.7336	.44283	304
I have noticed some white sticky deposits on my teeth	1.5230	.50029	304
I think that I cannot help having false teeth when I am old	1.4145	.49344	304
I think my teeth are getting worse despite my daily brushing	1.4737	.50013	304
I brush each of my teeth carefully	1.3454	.47628	304
I have never been professionally taught how to brush	1.7500	.43373	304
I think I can clean my teeth without using toothpaste	1.7829	.41295	304
I often check my teeth in a mirror after brushing	1.3388	.47409	304
It is impossible to prevent gum disease with toothbrushing alone	1.6645	.47295	304
I put off going to the dentist until I have a toothache	1.7401	.43929	304
I have used a dye to see how clean my teeth are	1.4375	.49690	304
I feel I sometimes take too much time to brush my teeth	1.7599	.42787	304

Supplementary Table S2. Item-total statistics of the HU-DBI questionnaire

Item-Total Statistics					
	Scale Mean if Item Deleted	Scale Variance if Item Deleted	Corrected Item-Total Correlation	Squared Multiple Correlation	Cronbach's Alpha if Item Deleted
My gums tend to bleed when I brush my teeth	17.2303	9.102	.382	.343	.810
I have noticed some white sticky deposits on my teeth	17.4408	8.591	.504	.418	.800
I think that I cannot help having false teeth when I am old	17.5493	8.664	.486	.268	.801
I think my teeth are getting worse despite my daily brushing	17.4901	8.482	.545	.419	.796
I brush each of my teeth carefully	17.6184	8.818	.451	.569	.804
I have never been professionally taught how to brush	17.2138	9.192	.357	.345	.812
I think I can clean my teeth without using toothpaste	17.1809	8.868	.520	.494	.799
I often check my teeth in a mirror after brushing	17.6250	8.704	.497	.599	.800
It is impossible to prevent gum disease with toothbrushing alone	17.2993	8.719	.493	.539	.801
I put off going to the dentist until I have a toothache	17.2237	9.065	.401	.491	.808
I have used a dye to see how clean my teeth are	17.5263	8.712	.464	.538	.803
I feel I sometimes take too much time to brush my teeth	17.2039	8.869	.497	.507	.801

Supplementary Table S3. Total variance explained in the exploratory factor analysis of the HU-DBI questionnaire

**Total Variance Explained**						
Component	Initial Eigenvalues			Rotation Sums of Squared Loadings		
	Total	% of Variance	Cumulative %	Total	% of Variance	Cumulative %
1	4.005	33.376	33.376	2.371	19.757	19.757
2	1.763	14.688	48.064	2.109	17.575	37.332
3	1.369	11.410	59.474	1.996	16.634	53.966
4	1.074	8.951	68.425	1.735	14.458	68.425
5	.793	6.605	75.030			
6	.688	5.730	80.760			
7	.607	5.057	85.817			
8	.500	4.163	89.979			
9	.419	3.495	93.475			
10	.307	2.562	96.037			
11	.260	2.169	98.206			
12	.215	1.794	100.000			
Extraction Method: Principal Component Analysis.						

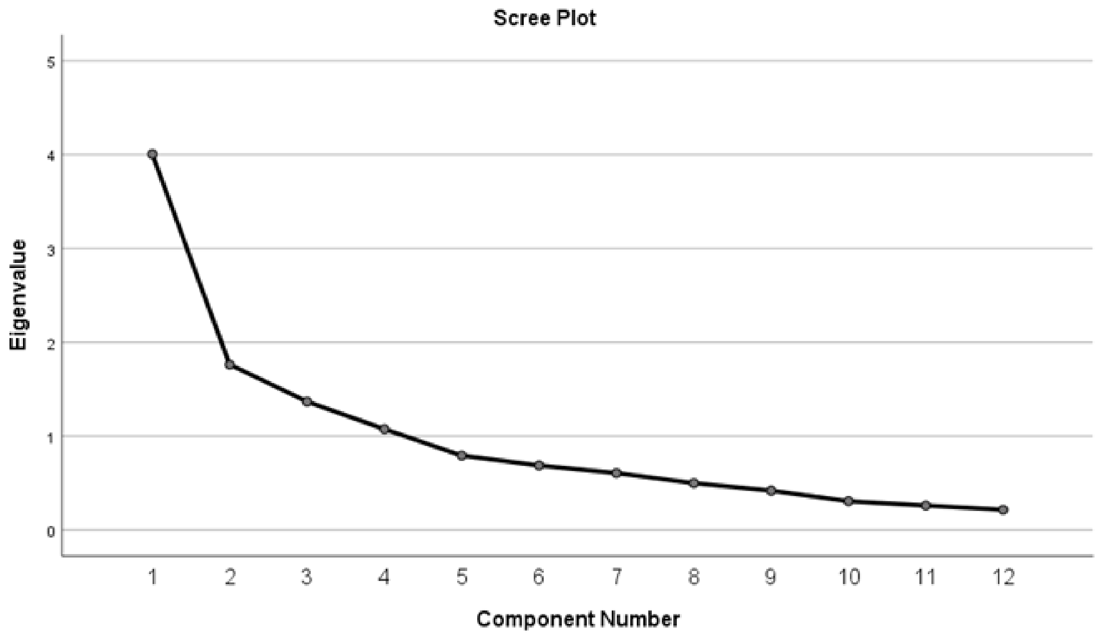

Supplementary Figure S1. Scree plot for the exploratory factor analysis of the HU-DBI questionnaire

Supplementary Table S4. Rotated component matrix of the HU-DBI questionnaire

**Rotated Component Matrix**[a]				
	Component			
	1	2	3	4
My gums tend to bleed when I brush my teeth	-.165	.486	.532	.161
I have noticed some white sticky deposits on my teeth	.249	.731	-.025	.198
I think that I cannot help having false teeth when I am old	.363	.382	.264	.154
I think my teeth are getting worse despite my daily brushing	.341	.651	-.008	.253
I brush each of my teeth carefully	.754	.225	-.231	.302
I have never been professionally taught how to brush	-.139	.673	.338	.030
I think I can clean my teeth without using toothpaste	.059	.334	.147	.772
I often check my teeth in a mirror after brushing	.866	.194	.096	-.043
It is impossible to prevent gum disease with toothbrushing alone	.295	.255	.782	-.121
I put off going to the dentist until I have a toothache	.030	-.014	.812	.308
I have used a dye to see how clean my teeth are	.748	-.196	.403	.221
I feel I sometimes take too much time to brush my teeth	.211	.106	.108	.856
Extraction Method: Principal Component Analysis. Rotation Method: Varimax with Kaiser Normalization.				
a. Rotation converged in 14 iterations.				
